# Synthesis and Antimicrobial Activity of Novel Substituted Ethyl 2-(Quinolin-4-yl)-propanoates

**DOI:** 10.3390/molecules18033227

**Published:** 2013-03-13

**Authors:** M. Akram Khan, Keith Miller, Kim Drummond Rainsford, Yong Zhou

**Affiliations:** Biomedical Research Centre, Sheffield Hallam University, Howard Street, Sheffield, S1 1WB, UK; E-Mails: m.a.khan@shu.ac.uk (M.A.K.); k.miller@shu.ac.uk (K.M.); k.d.rainsford@shu.ac.uk (K.D.R.); yongzhou603@yahoo.com.cn (Y.Z.)

**Keywords:** synthesis, 4**-**quinolylpropionic esters, Gould-Jacobs reaction, anti-microbial activity

## Abstract

Substituted 4-hydroxyquinolines were synthesized from anilines and diethyl 2-(ethoxymethylene)malonate by the Gould-Jacobs reaction via cyclization of the intermediate anilinomethylenemalonate followed by hydrolysis and decarboxylation. The 4-hydroxyquinolines reacted with phosphorous oxychloride to form 4-chloroquinolines, which reacted on heating with diethyl sodiomethylmalonate in DMF to yield moderate yields of substituted ethyl 2-(quinolin-4-yl)propanoates, many of which showed potent antimicrobial activity against *Helicobacter* *pylori*.

## 1. Introduction

Many natural products in the plant kingdom contain the quinoline nucleus, such as quinine, which has well known anti-malarial and anti-inflammatory activities [1,2]. During World War II an enormous effort was realised by synthetic organic chemists to develop new anti-malarials based on quinine as the supply of quinine from natural sources was insufficient for the Allied and US forces fighting in South East Asia. This intense synthetic attention resulted in a large number of substituted 4-chloroquinolines which were subsequently reacted with a variety of alkylamines to form 4-alkylaminoquinolines as substitution products [3,4,5,6], from which the new potent synthetic anti-malarial chloroquin resulted. Quinoline compounds have also had a history of being potential anti-inflammatory compounds [7,8,9].

In the last twenty five years there has been a steep decline in the commercial output and research and development of antimicrobial agents by the major pharmaceutical companies due to the more attractive commercial returns that can be made for treatments of chronic human diseases. At the same time there has been an explosion both in the numbers of pathogenic bacteria that have become resistant to antibiotics and of immuno-compromised patients that are particularly susceptible to opportunistic pathogens. More recently there has also been rapid emergence of new resistance mechanisms to antibiotics recently introduced to the market, including linezolid and daptomycin. The discovery of new therapeutic agents with novel modes of action and no cross-resistance with current antibiotics will be vital to meet the threats created by the emergence of bacteria resistant to current therapeutic agents [10].

*Helicobacter pylori* (formerly *Campylobacter pylori*) and its clinical association with the development of peptic ulcers has led to the development of various chemotherapeutic agents to eliminate infection caused by this pathogen [11,12,13,14,15]. *H. pylori* is also implicated in the development of acute and chronic gastritis, gastric adenocarcinoma and gastric lymphoma (MALT), and has been classified as a class I carcinogen in humans and is a major contributing factor in the development of gastric cancer [16]. Infection with *H. pylori* is treated with a combination of clartithromycin, ampicillin and a proton pump inhibitor, but this triple therapy approach is costly [17]. The infection is typically eradicated in up to 90% of patients but side effects, poor compliance and the development of antimicrobial resistance are common causes of treatment failure [18]. *H*. *pylori* infection has been implicated with increased COX-2 expression in gastric antral mucosa for both NSAID users and non-users [19,20,21]. In this paper we report a set of novel quinoline derived propionic acid esters **10a**–**p** with *H*. *pylori* activity. These are novel compounds that to the best of our knowledge have not been made before.

## 2. Results and Discussion

### 2.1. Chemistry

**Scheme 1:**
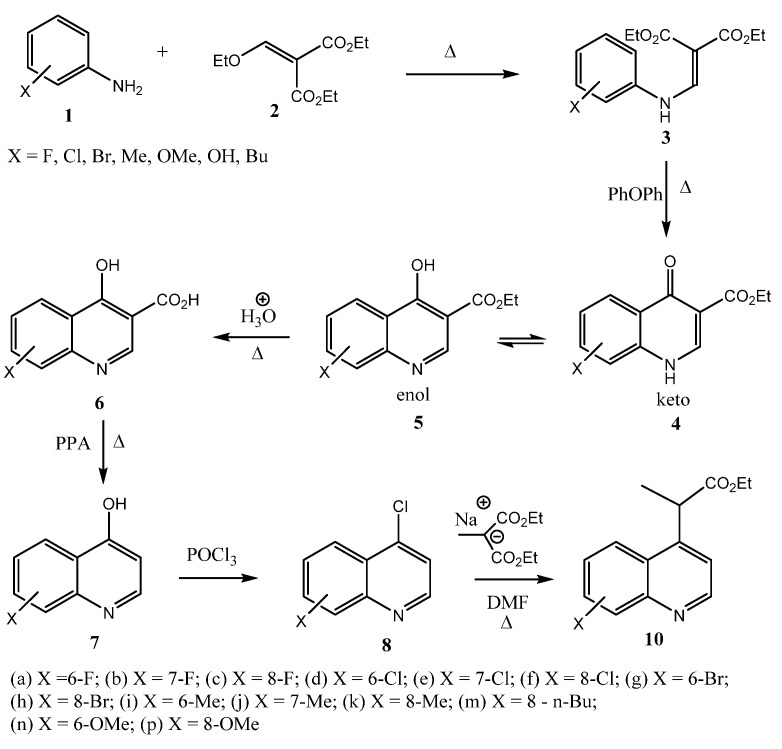
Synthesis of 4-chloroquinolines and ethyl 2-(quinolin-4-yl)propanoates.

**Scheme 2:**
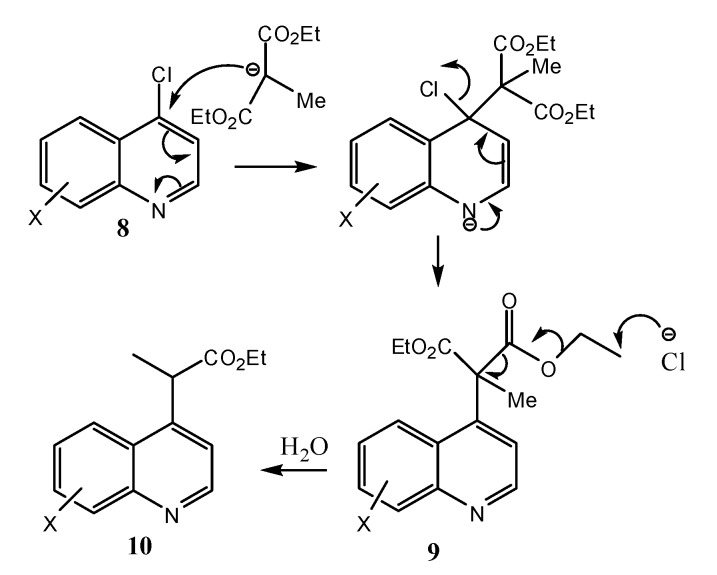
Mechanism of the de-ethoxycarbonylation by the Krapcho reaction.

Classically a variety of methods have existed for the synthesis of quinolines [22]. In this work we have used the Gould-Jacobs reaction [23,24,25] of anilines **1** and diethyl 2-(ethoxymethylene) malonate (**2**) for the synthesis of 4-hydroxyquinolines **7** as key intermediates (Scheme 1). Thus, commercially available substituted anilines **1** were condensed with **2** to yield the anilinodiesters **3** in high yields. Compounds **3** underwent a thermally induced intramolecular cyclisation in diphenyl ether to form the quinolones **4** in high yields. Hydrolysis followed by thermal decarboxylation of enols **5** furnished the requisite 4-hydroxyquinolines **7** in good yields. The 4-chloroquinolines **8**, obtained from **7** by reaction with phosphorus oxychloride [26], underwent nucleophilic aromatic substitution reactions with diethyl sodiomethylmalonate in DMF to yield products **9** which subsequently de-ethoxycarbonylated by the Krapcho reaction [27] to produce the target propionic esters **10** in moderate to good yields (Scheme 2).

Although nucleophilic substitution reactions of halopyridines and haloquinolines with a variety of nucleophiles are well documented [28], this is the first time such a reaction has been reported on substituted 4-chloroquinolines to form substituted ethyl 2-(quinolin-4-yl)propanoates. A literature search showed that ethyl 2-(quinilin-4-yl)propanoate (**10**, X = H) has been reported [29]. Synthesis of a pyridine equivalent ethyl 2-(4-pyridyl)propionate (**11**) by titanium enolate addition to the 4-position of 1-phenoxycarbonylpyridinium salts to give 1,4-dihydropyridines which on subsequent aromatization provided 4-(2-oxoalkyl) pyridines has been reported [30]. A 3-substituted(4-pyridyl) propionic acid **12** has also been reported as a key intermediate in the synthesis of the potent and long-acting histamine H_2_-receptor antagonist SK&F 93574 [31].

**Scheme 3:**
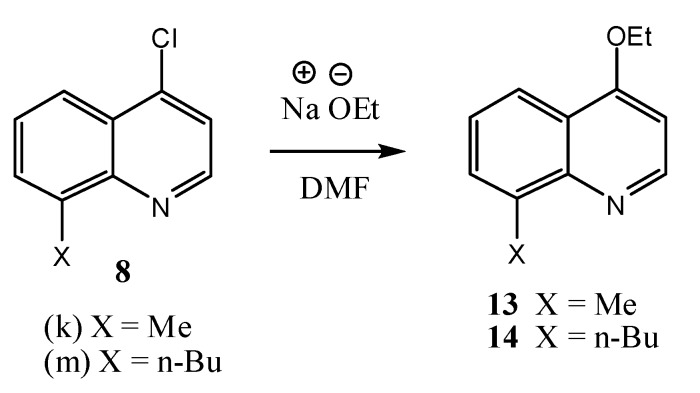
Formation of side products **13** and **14** during the synthesis of **10k** and **10m**.

During the chromatographic purification of the two compounds **10k** and **10m** we isolated the compounds 4-ethoxyquinolines **13** and **14** as side products. 4-Ethoxyquinolines have previously been reported as side products in the preparation of ethyl 7-chloro-4-hydroxyquinoline-3-carboxylate (**4e**) from diethyl ethoxymethylmalonate (**2**) and 3-chloroaniline [32]. One possible explanation is that in our case sodium hydroxide had formed in small amounts when we were adding sodium hydride to the DMF as a result of some moisture in the DMF and this caused partial hydrlolysis of diethyl methylmalonate to form ethanol. The ethanol reacted with sodium hydride to form sodium ethoxide which then produced the 4-ethoxyquinolines **13** and **14** by nucleophilic substitution reactions (Scheme 3).

All the new compounds **10a**–**p**, **13** and **14** were fully characterized and purity check on the compounds was performed using GC-MS.

### 2.2. Microbiological Results and Discussion

All of the quinoline compounds **10b**–**p** exhibited antimicrobial effects against our panel of organisms (Table 1).

**Table 1:** Minimum inhibitory concentrations (MIC) of novel substituted ethyl 2-(quinolin-4-yl) propanoates for a range of microorganisms.

	MIC (µg/mL)							
	Gram positive bacteria			Gram negative bacteria				Fungi
**Inhibitor**	SA	SE	BS	EC	PA	KP	HP	CA
**10b**	64	512	512	16	64	>512	8	128
**10c**	64	>512	>512	32	64	>512	32	>512
**10e**	128	256	512	64	64	512	16	256
**10f**	64	512	512	16	128	>512	32	256
**10g**	64	256	512	64	64	512	16	256
**10i**	128	256	512	16	16	512	64	256
**10j**	128	512	512	64	128	512	32	256
**10k**	16	512	256	64	64	512	32	128
**10n**	32	512	512	16	64	512	64	256
**10p**	128	>512	64	64	128	>512	16	256
Ampicillin	0.032	0.032	8	4	>512	32	2	ND
Clarithromycin	0.5	0.5	ND	ND	ND	ND	0.25	ND
Fluconazole	ND	ND	ND	ND	ND	ND	ND	1
Tetracycline	2	2	8	1	32	2	8	512

SA: Staphylococcus aureus, EF: Enterococcus faecalis, SE: Staphylococcus epidermidis, BS: Bacillus subtilis, EC: Escherichia coli, PA: Pseudomonas aeruginosa, KP: Klebsiella pneumoniae, HP: Helicobacter pylori, CA: Candida albicans, ND: not determined.

**Figure 1:**
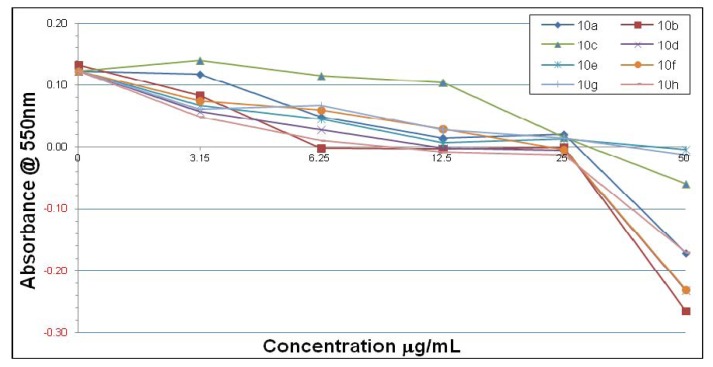
Inhibitory effects of compounds **10a**–**h** on *H. pylori* 3339 after 7 days.

**Figure 2:**
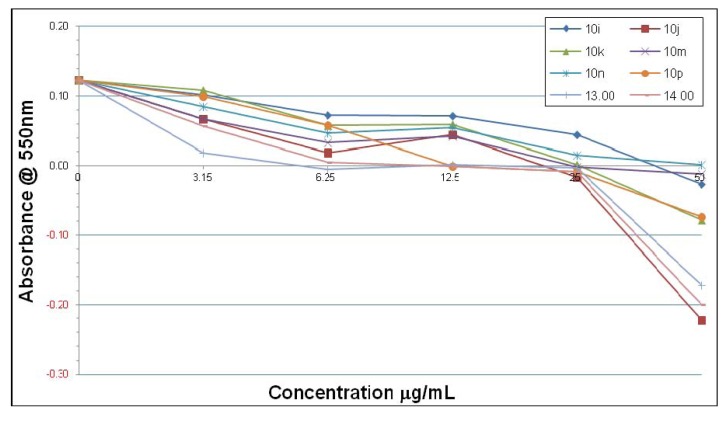
Inhibitory effects of compounds **10i**–**p**, **13** and **14** on *H. pylori* 3339 after 7 days.

There was modest antimicrobial activity against *H. pylori* when compared with the standard anti-*Helicobacter* agents, however, there appears to be no Gram-specificity which makes these agents promising for broad-spectrum anti-infective development. There is quite a significant contrast in inhibitory activity for the different quinolines using the two different types of *H. pylori* strains. Thus, in Figure 1 and Figure 2 it can be seen that overall many of the compounds have inhibitory effects, but there are some differences in the concentration-responses for the inhibition of *H. pylori* strain 3339 obtained with compounds **10a**–**p**, **13** and **14** after 7 days. Compounds **10b**, **13** and **14** show relatively potent inhibition in the low concentration range 3–6 μmol/mL toward *H. pylori* 3339, whilst compounds **10e**, **10g** and **10n** were the least potent at both low (3–6 μg/mL) and high concentrations (25–50 μg/mL). The decline in absorbance at concentrations of 25–50 µg/mL below zero probably represents the lysis of the cells by many of the compounds.

**Figure 3:**
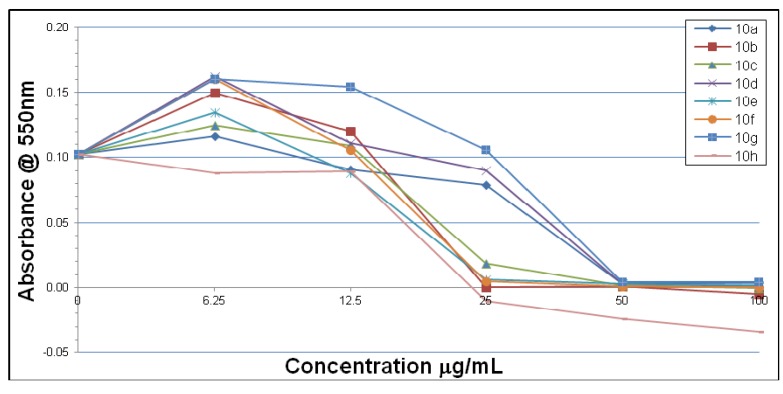
Inhibitory effects of compounds **10a**–**h** on *H. pylori* 26695 after 7 days.

**Figure 4:**
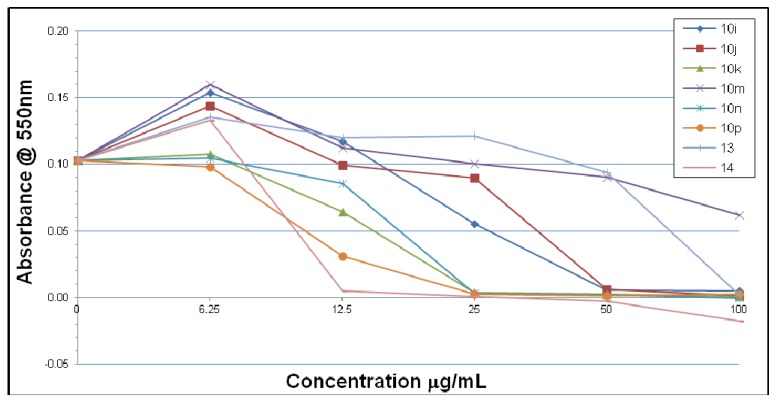
Inhibitory effects of compounds **10i**–**p**, **13** and **14** on *H. pylori* 26695 after 7 days.

In contrast to the situation with *H. pylori* strain 3339, the results with strain 26695 were somewhat different. Thus, only compounds **10h** and **10p** show inhibitory activity at low concentration (6.25 μg/mL) while compounds **10g**, **10m** and **13** failed to show any inhibitory effects (Figure 3 and Figure 4). However, compounds **10p** and **14** show a dramatic potency in the concentration range 6-12 μmol/mL. At the concentration of 25 μg/mL all of the four compounds **10k**, **10m**, **10p** and **14** show equal and complete inhibitory activity.

In the context of quantitative structure activity relationship (QSAR) the following conclusions can be made. An alkyl group in positions 6, 7 and 8 on the quinoline ring leads to lack of reactivity as shown by the reactivity profile of compounds **10i**, **10j** and **10m** on *H. pylori* 26695 at 25 μg/mL concentration. A methyl group in the position-6 of quinoline showed lack of potency against *H. pylori* 3339 at 25 μg/mL concentration, as uniquely illustrated by compound **10i**. However, an alkyl group in position-8 such as a methyl or n-butyl group with an alkoxy group such as ethoxy seems to enhance potency, as shown by compounds 13 and 14 against *H.* *pylori* 3339 at low concentration. Furthermore, the presence of a halogen atom in position-6 caused lack of reactivity against *H. pylori* 26695 at 25 μg/mL concentration as shown by compounds **10a**, **10d** and **10g**.

## 3. Experimental

### 3.1. Chemistry

Melting points were recorded on Stuart SMP3 digital apparatus; ^1^H-NMR and ^13^C-NMR spectra were recorded in CDCl_3_ on a Bruker AC 250 MHz and a Bruker Avance III 400 MHz spectrometer, respectively. GC-MS analyses of quinoline derivatives **10a**–**p** were performed with an Alignment Technology 5975C VLMSD mass spectrometer interfaced with a GC7890A gas chromatography system with triple-axis detector having an auto-sampler set-up system and a splitless injection (inlet temperature 250 °C) HP5MS capillary column (30 m, 0.25 mm i.d., 0.25 um film thickness) was selected. The oven temperature was programmed from 150 °C (initial hold time of 1 min) to 280 °C at a rate of 10–20 °C min^−^^1^; this final temperature was maintained for 15 min. Mass spectra (MS) were obtained on VG 770E spectrometer operated in EI mode at 70 eV. High resolution accurate mass (HRMS) of compounds was detected using an Applied Biosystems/MDS Sciex Hybrid quadrupole time-of-flight instrument (Q-Star Pulsar-i) fitted with an orthogonal MALDI ion source and an ND: VAG Laser. TLC analyses were done using Merck silica gel coated aluminium sheets and flash chromatography was performed using BDH flash silica gel and the eluents are indicated in parenthesis for each compound. The two commonly used eluents are abbreviated: ethyl acetate (EtOAc), petroleum ether (Pet.).

We used the exact procedures in the literature to synthesise all the 4-hydroxyquinolines **7** [24] (Scheme 1) and for the conversion of 4-hydroxyquinolines **7** to 4-chloroquinolines **8** we used another reported procedure [26].

*General synthetic procedure, exemplified by ethyl 2-(7-chloroquinolin-4-yl)propanoate* **(10e**). Sodium hydride (3.0 g, 60% dispersion in mineral oil, 73.8 mmol) was slowly added in portions to a magnetically stirred solution of diethyl methylmalonate (12.80 g, 73.8 mmol) in dry dimethylformamide (DMF) (55 mL). After the vigorous exothermic reaction had subsided 4,7-dichloroquinoline (4.87 g, 24.6 mmol) was added and the stirred reaction mixture was refluxed in an oil bath for 24 h. The solvent DMF was removed by distillation under reduced pressure and to the residue was added water (100 mL) and extracted with dichloromethane (2 × 120 mL). The combined organic layer, after drying over anhydrous magnesium sulphate, was filtered and evaporated to yield an oily residue which was purified by flash column chromatographed (1:3, EtOAc:pet. ether) to yield the pure product **10a** as a colourless thick oil (5.84 g, 90% yield); t_R_ 11.209 min (100%); IR ν 1731 (C=O) cm^−1^; ^1^H-NMR: δ_Η_ 1.17 (3Η, t, *J*_HH_ 7.2 Hz, -OCH_2_-CH_3_), 1.66 (3Η, d, *J*_HH_ 7.2 Hz , >CH-CH_3_), 4.14 (2H, qd, *J*_HH_ 7.2 and 1.0 Hz, -O-CH_2_-), 4.45 (1H, q, *J*_HH_ 7.2 Hz, >CH-), 7.38 (1H, d, *J*_HH_ 4.6 Hz, H-3), 7.53 (1H, dd, *J*_HH_ 7.8 Hz and 1.9 Hz, H-6), 8.02 (1H, d, *J*_HH_ 7.8 Hz, H-5), 8.15 (1H, d, *J*_HH_ 1.9 Hz, H-8), 8.88 (1H, d, *J*_HH_ 4.6 Hz, H-2); ^13^C-NMR: δ_C_ 13.96 (CH_3_), 17.52 (CH_3_), 41.03 (OCH_2_), 61.32 (>CH-), 119.38 (3C), 124.55 (5C), 125.19 (10C), 127.69 (6C), 129.00 (8C), 135.08 (7C), 146.53 (4C), 148.84 (9C), 151.27 (2C), 173.13 (C=O); MS (EI) *m/z* 263.5 (M^+^, 35%), 235.5 (McLafferty ion, M−CH_2_=CH_2_, 1.5%), 190.5 (M−CO_2_Et, 100%), 162.5 (M−CH_3_CHCO_2_Et, 22%). HRMS: Found 263.0802 (Cl^35^) and 265.0790 (Cl^37^). Calcd. for C_14_H_15_NO_2_Cl 264.0791 (Cl^35^) and 266.0762 (Cl^37^).

The following compounds were prepared as above and purified by flash column chromatography (1:3, EtOAc:pet. ether).

*Ethyl 2-(6-fluoroquinolin-4-yl)propanoate* (**10a**). Colourless oil, (27% purified yield); t_R_ 11.591 min (97.637%); ν 1728 (C=O) cm^−1^; ^1^H-NMR: δ_Η_ 1.11 (3Η, t, *J*_HH_ 7.2 Hz, -OCH_2_-CH_3_), 1.61 (3Η, d, *J*_HH_ 7.2 Hz, >CH-CH_3_), 4.13 (2H, qd, *J*_HH_ 7.2 and 1.0 Hz, -O-CH_2_-), 4.35 (1H, q, *J*_HH_ 7.2 Hz, >CH-), 7.27–7.43 (3H, m, H-3, H-5 and H-7), 8.00 (1H, d, *J*_HH_ 4.0 Hz, H-8), 8.76 (1H, d, *J*_HH_ 4.5 Hz, H-2); ^13^C-NMR: δ_C_ 14.01(CH_3_), 17.57 (OCH_2_), 41.34 (>C=Ο), 61.38 (>CΗ−), 107.87 (8C), 120.22 (6C), 121.42 (3C), 127.04 (10C), 128.71 (5C), 144.63 (4C), 146.34 (9C), 148.85 (2C), 160.36 (7C), 173.18(C=O); MS (EI) *m/z* 247.2 (M^+^, 35%), 174.2 (M−CO_2_Et, 100%); HRMS (MALDI): Found 248.1096 Calcd. for C_14_H_15_NO_2_F 248.1087.

*Ethyl 2-(7-fluoroquinolin-4-yl)propanoate* (**10b)**. Colourless oil, (38% purified yield); t_R_ 10.123 min (96.926%); ν 1731 (C=O) cm^−1^; ^1^H-NMR: δ_H_ 1.12 (3Η, t, *J*_HH_ 7.3 Hz, -OCH_2_-CH_3_ ), 1.62 (3Η, d, *J*_HH_ 7.0 Hz, >CH-CH_3_), 4.12 (2H, q, *J*_HH_ 7.3 Hz, -O-CH_2_-), 4.41 (1H, q, *J*_HH_ 7.0 Hz, >CH-), 7.27–7.37 (2H, m, H-3 and H-6), 7.72 (1H, dd, *J* = 7.0 and 2.7 Hz, H-8), 8.06 (1H, dd, *J*_HH_ 6.1 and 3.3 Hz, H-5), 8.83 (1H, d, *J*_HH_ 4.6 Hz, H-2); ^13^C-NMR: δ_C_ 13.97 (CH_3_), 17.60 (CH_3_), 41.32 (>CH-), 61.34 (OCH_2_), 173.23 (C=O); MS (EI) *m/z* 247.2 (M^+^, 31%), 202.2 (M−OC_2_H_5_, 6%) 174.2 (M−CO_2_Et, 100%); HRMS: Found 248.1083 Calcd. for C_14_H_15_NO_2_F 248.1087.

*Ethyl 2-(8-fluoroquinolin-4-yl)propanoate* (**10c**). Colourless oil, (37% purified yield); t_R_ 11.789 min (100%); ν 1729 (C=O) cm^−1^; ^1^H-NMR: δ_H_ 1.14 (3H, t, *J*_HH_ 7.2 Hz, -OCH_2_-CH_3_), 1.67 (3Η, d, *J*_HH_ 7.2 Hz, >CH-CH_3_), 4.14 (2H, qd, *J*_HH_ 7.2 and 1.0 Hz, -O-CH_2_-), 4.5 (1H, q, *J*_HH_ 7.2 Hz, >CH-), 7.41 (2H, m, H-7), 7.46 (1H, d, *J*_HH_ 4.5 Hz, H-3), 7.53 (1H, dt, *J*_HH_ 7.9 and 5.3 Hz, H-6), 7.87 (1H, d, *J*_HH_ 8.5Hz, H-5), 8.92 (1H, d, *J*_HH_ 4.5 Hz, H-2); ^13^C-NMR: δ_C_ 13.98 (CH_3_), 17.58 (CΗ_3_), 41.34 (>CΗ-), 61.36 (ΟCΗ_2_), 113.25 (7C), 118.83 (5C), 120.14 (3C), 126.48 (6C), 128.44 (10C), 138.85 (9C), 146.38 (4C), 150.34 (2C), 158.53 (8C), 173.20 (C=Ο); MS (EI) *m/z* 247.2 (M^+^, 41%), 202.2 (M−OC_2_H_5_, 8%) 174.2 (M−CO_2_Et, 100%); HRMS: Found 247.1090 Calcd. for C_14_H_15_NO_2_F 248.1087.

*Ethyl 2-(6-chloroquinolin-4-yl)propanoate* (**10d**). Colourless oil, (78% purified yield); t_R_ 10.996 min (100%); ν 1731 (>C=O) cm^−1^; ^1^H-NMR: δ_H_ 1.19 (3Η,t, *J*_HH_ 7.0 Hz, -OCH_2_-CH_3_), 1.65 (3Η, d, *J*_HH_ 7.0 Hz, >CH-CH_3_), 4.16 (2H, q, *J*_HH_ 7.3 Hz, -O-CH_2_-), 4.40 (1H, q, *J*_HH_ 7.0 Hz, >CH), 7.40 (1H, d, *J*_HH_ 4.6 Hz, H-3), 7.65 (1H dd, *J*_HH_ 6.7 and 2.1 Hz H-7), 8.06 (1H, d, *J*_HH_ 2.1 Hz, H-6), 8.08 (1H, d, *J*_HH_ 6.7 Hz, H-8), 8.92 (1H, d, *J*_HH_ 4.6 Hz, H-2); ^13^C-NMR: δ_C_ 13.96 (CH_3_), 17.52 (CH_3_), 41.03 (>CH-), 61.32 (OCH_2_), 119.38 (5C), 124.55 (3C), 125.19 (10C), 127.69 (8C), 129.00 (7C), 135.08 (6C), 146.53 (4C), 148.84 (9C), 151.27 (2C), 173.13 (C=O); MS (EI) *m/z* 263.5 (M^+^, 35%), 235.5 (McLafferty ion, M−CH_2_=CH_2_, 1.5%), 190.5 (M−CO_2_Et, 100%), 162.5 (M−CH_3_CHCO_2_Et, 22%). HRMS: Found 263.0786 (Cl^35^) and 265.0770 (Cl^37^). Calcd. for C_14_H_15_NO_2_Cl 264.0791 (Cl^35^) and 265.0762 (Cl^37^).

*Ethyl 2-(8-chloroquinolin-4-yl)propanoate* (**10f**). Colourless oil, (72% purified yield); t_R_ 10.593 min (100%); ν 1730 (C=O) cm^−1^; ^1^H-NMR: δ_H_ 1.15 (3Η,t, *J*_HH_ 7.3 Hz, -OCH_2_-CH_3_), 1.66 (3Η, d, *J*_HH_ 7.3 Hz, >CH-CH_3_), 4.14 (2H, q, *J*_HH_ 7.3 Hz, -O-CH_2_-), 4.48 (1H, q, *J*_HH_ 7.3 Hz, >CH-), 7.46 (1H, d, *J*_HH_ 4.6 Hz, H-3), 7.48 (1H t, *J*_HH_ 7.5 Hz, H-6), 7.81 (1H, dd, *J*_HH_ 7.5 and 1.2 Hz, H-7), 8.02 (1H, dd, *J*_HH_ 7.5 and 1.2 Hz, H-5), 9.01 (1H, d, *J*_HH_ 4.6 Hz, H-2); ^13^C-NMR: δ_C_ 13.93 (CH_3_), 17.62 (CH_3_), 41.18 (>CH-), 61.26 (OCH_2_), 119.99 (5C), 122.19 (3C), 126.51 (6C), 128.11 (10C), 129.45 (7C),134.32 (8C), 144.61 (4C), 146.93 (9C), 150.63 (2C), 173.07 (C=O); MS (EI) *m/z* 263.5 (M^+^, 29%), 190.5 (M−CO_2_Et, 55%), 170.5 (M−CH_3_CHCO_2_Et, 100%). HRMS: Found 264.0788 (Cl^35^) and 265.0777 (Cl^37^). Calcd. for C_14_H_15_NO_2_Cl 264.0791 (Cl^35^) and 266.0762 (Cl^37^).

*Ethyl 2-(6-bromoquinolin-4-yl)propanoate* (**10g**). Colourless oil, (38% purified yield); t_R_ 11.483 min (97.207%); ν 1718 (C=O) cm^−1^; ^1^H-NMR: δ_H_ 1.20 (3H, t, *J*_HH_ 7.5 Hz, -OCH_2_-CH_3_), 1.65 (3Η, d, *J*_HH_ 7.2 Hz, >CH-CH_3_), 4.16 (2H, q, *J*_HH_ 7.5 Hz, -O-CH_2_-), 4.42 (1H, q, *J*_HH_ 7.2 Hz, >CH-), 7.40 (1H, d, *J*_HH_ 4.5 Hz, H-3), 7.77 (1H dd, *J*_HH_ 6.8 and 2.1 Hz H-7), 8.00 (1H, d, *J*_HH_ 9.3 Hz, H-8), 8.26 (1H, d, *J*_HH_ 2.4 Hz, H-5), 8.88 (1H, d, *J*_HH_ 4.5 Hz, H-2); ^13^C-NMR: δ_C_ 13.92 (CH_3_), 17.63 (CH_3_), 40.74 (>CH-), 61.36 (OCH_2_), 119.92 (6C), 121.08 (3C), 125.53 (5C), 127.77 (10C), 132.18 (8C), 132.47 (7C), 145.51 (4C), 147.03 (9C), 150.42 (2C), 173.05 (C=O); MS (EI) *m/z* 307/309 (M^+^, 18%), 234/236 (M−CO_2_Et, 26%), 154 [M−(Br+CO_2_Et), 22%]; HRMS: Found 308.0299 (Br^79^) and 310.0276 (Br^81^). Calcd. for C_14_H_15_NO_2_Br 308.0286 (Br^79^) and 310.0266 (Br^81^).

*Ethyl 2-(8-bromoquinolin-4-yl)propanoate* (**10h**). Colourless oil, (59% purified yield); t_R_ 12.192 min (97.219%); ν 1729 (C=O) cm^−1^; ^1^H-NMR: δ_H_ 1.14 (3Η,t, *J*_HH_ 7.5 Hz, -OCH_2_-CH_3_), 1.65 (3Η, d, *J*_HH_ 7.3 Hz, >CH-CH_3_), 4.13 (2H, q, *J*_HH_ 7.5 Hz, -O-CH_2_-), 4.48 (1H, q, *J*_HH_ 7.3 Hz, >CH-), 7.39 (1H, t, *J*_HH_ 7.5 Hz, H-6), 7.45 (1H, d, *J*_HH_ 4.5 Hz H-3), 8.02 (1H, d, *J*_HH_ 7.5 Hz, H-5), 8.06 (1H, d, *J*_HH_ 7.5 Hz, H-7), 9.00 (1H, d, *J*_HH_ 4.5 Hz, H-2); ^13^C-NMR: δ_C_ 13.97 (CH_3_), 17.65 (CH_3_), 41.08 (>CH-), 61.27 (OCH_2_), 119.99 (3C), 122.95 (5C), 125.80 (8C), 127.02 (6C), 128.05 (10C), 132.94 (7C), 145.33 (9C), 146.93 (4C), 150.86 (2C), 173.04 (C=O); MS (EI) *m/z* 307/309 (M^+^, 22%), 234/236 (M−CO_2_Et, 24%), 154 [M−(Br+CO_2_Et), 47%], 84 (100%); HRMS: Found 308.0288 (Br^79^) and 310.0265 (Br^81^). Calcd. for C_14_H_15_NO_2_Br 308.0286 (Br^79^) and 310.0266 (Br^81^).

*Ethyl 2-(6-methylquinolin-4-yl)propanoate* (**10i**). Colourless oil, (54% purified yield); t_R_ 10.727 min (97.943%); ν 1729 (C=O) cm^−1^; ^1^H-NMR: δ_H_ 1.17 (3Η, t, *J*_HH_ 7.2 Hz, -OCH_2_CH_3_ ), 1.63 (3Η, d, *J*_HH_ 7.2 Hz, >CH-CH_3_), 2.53 (3H, s, -CH_3_), 4.12 (2H, q, *J*_HH_ 7.2 Hz, -O-CH_2_-), 4.47 (1H, q, *J*_HH_ 7.2 Hz, >CH-), 7.32 (1H, d, *J*_HH_ 4.5 Hz, H-3), 7.50 (1H dd, *J*_HH_ 7.0 and 2.7 Hz, H-7), 7.83 (1H, d, *J*_HH_ 2.7 Hz, H-5), 8.03 (1H, d, *J*_HH_ 7.0 Hz, H-8), 8.79 (1H, d, *J*_HH_ 4.5 Hz, H-2); ^13^C-NMR: δ_C_ 13.80 (CH_3_), 17.69 (CH_3_), 21.92 (CH_3_), 40.79 (>CH-), 61.09 (OCH_2_), 119.00 (3C), 121.85 (5C), 126.55 (10C), 130.17 (8C), 131.14 (7C), 136.60 (6C), 145.59 (4C), 146.99 (9C), 149.25 (2C), 173.56 (C=O); MS (EI) *m/z* 243.3 (M^+^, 65%), 215.2 (McLafferty ion, M−CH_2_=CH_2_, 5%), 170.2 (M−CO_2_Et, 70%); HRMS: Found 244.1327. Calcd. for C_15_H_18_NO_2_ 244.1337.

*Ethyl 2-(7-methylquinolin-4-yl)propanoate* (**10j**). Colourless oil, (58% purified yield); t_R_ 10.886 min (100%); ν 1729 (C=O) cm^−1^; ^1^H-NMR: δ_H_ 1.14 (3H, t, *J*_HH_ 7.0 Hz, CH_2_CH_3_), 1.63 (3Η, d, *J*_HH_ 7.0 Hz, >CH-CH_3_), 2.53 (3H, s, -CH_3_), 4.15 (2H, q, *J*_HH_ 7.0 Hz, -O-CH_2_-), 4.49 (1H, q, *J*_HH_ 7.0 Hz, >CH-), 7.26 (1H, d, *J*_HH_ 4.5 Hz, H-3), 7.41 (1H dd, *J*_HH_ 7.0 and 2.7 Hz H-6), 7.92 (1H, d, *J*_HH_ 2.7 Hz, H-8), 7.96 (1H, d, *J*_HH_ 7.0 Hz, H-5), 8.83 (1H, d, *J*_HH_ 4.5 Hz, H-2); ^13^C-NMR: δ_C_ 13.99 (CH_3_), 17.66 (CH_3_), 21.57 (CH_3_ at 7C), 41.03 (>CH-), 61.19 (OCH_2_), 118.35(3C), 122.70 (5C), 124.71 (10C), 129.01 (6C),129.10 (8C), 139.43 (7C), 146.19 (4C), 148.69 (9C), 150.21 (2C), 173.56 (C=O); MS *m/z* 243.3 (M^+^, 63%), 215.2 (McLafferty ion, M−CH_2_=CH_2_, 7%), 170.2 (M−CO_2_Et, 65%); HRMS: Found 244.1331. Calcd. for C_15_H_18_NO_2_ 244.1337.

*Ethyl 2-(8-methylquinolin-4-yl)propanoate* (**10k**). Colourless oil, (59% purified yield); t_R_ 9.451 min (100%);ν 1731 (C=O) cm^−1^; ^1^H-NMR: δ_H_ 1.16 (3H, t, *J*_HH_ 7.0 Hz, CH_2_CH_3_), 1.64 (3Η, d, *J*_HH_ 7.0 Hz, >CH-CH_3_), 2.82 (3H, s, -CH_3_), 4.16 (2H, q, *J*_HH_ 7.0 Hz, -O-CH_2_-), 4.46 (1H, q, *J*_HH_ 7.0 Hz, >CH-), 7.36 (1H, d, *J*_HH_ 4.1 Hz, H-3), 7.46 (1H t, *J*_HH_ 6.7 Hz H-6), 7.57 (1H, d, *J*_HH_ 6.7 Hz, H-7), 7.93 (1H, d, *J*_HH_ 6.7 Hz, H-5), 8.0 (1H, d, *J*_HH_ 4.1 Hz, H-2); ^13^C-NMR: δ_C_ 13.97 (CH_3_), 17.65 (CH_3_), 21.57 (CH_3_), 41.03 (>CH-), 61.22 (OCH_2_), 118.55 (3C), 122.68 (5C), 125.11 (6C), 129.90 (10C), 129.97 (7C), 136.32 (8C), 146.19 (4C), 148.20 (9C), 150.10 (2C), 173.56 (C=O); MS (EI) *m/z* 243.3 (M^+^, 67%), 215.2 (McLafferty ion, M−CH_2_=CH_2_, 8%), 170.2 (M−CO_2_Et, 69%); HRMS: Found 244.1335. Calcd. for C_15_H_18_NO_2_ 244.1337.

*Ethyl 2-(8-but-1-ylquinolin-4-yl)propanoate* (**10m**). Colourless oil, (38% purified yield); t_R_ 11.844min (100%); ν 1732 (C=O) cm^−1^; ^1^H-NMR: δ_H_ 0.88 (3Η, t, *J*_HH_ 7.0 Hz, −CΗ_2_−CΗ_3_), 1.16 (3Η, t, *J*_HH_ 7.0 Hz, -OCH_2_-CH_3_), 1.55 (2Η, sextet, -CH_2_-CH_2_-CH_3_), 1.65 (3Η, d, *J*_HH_ 7.0 Hz, >CH-CH_3_), 1.83 (2H, quintet, -CH_2_-CH_2_-CH_2_-), 3.29 (2H, t, *J*_HH_ 7.0 Hz, Ar-CH_2_-CH_2_-), 4.14 (2H, q, *J*_HH_ 7.0 Hz, -O-CH_2_-), 4.47 (1H, q, *J*_HH_ 7.0 Hz, >CH-), 7.34 (1H, d, *J*_HH_ 4.3 Hz, H-3), 7.49 (1H, t, *J*_HH_ 7.0 Hz, H-6), 7.55 (1H, dd, *J*_HH_ 7.0 and 1.5 Hz, H-7), 7.93 (1H, dd, *J*_HH_ 7.0 and 1.5 Hz, H-5), 8.90 (1H, d, *J*_HH_ 4.3 Hz, H-2); ^13^C-NMR: δ_C_ 13.97 (CH_3_), 14.16 (CH_3_), 17.65 (CH_3_), 22.84 (CH_2_), 31.46 (CH_2_), 32.82 (CH_2_), 41.05 (>CH-), 61.32 (OCH_2_), 119.67 (3C), 121.53 (5C), 125.14 (6C), 128.95 (10C), 135.42 (8C), 140.96 (7C), 147.86 (4C), 150.08 (2C), 161.73 (9C), 173.46 (C=O); MS (EI) *m/z* 285 (M^+^, 26%), 212 (M−CO_2_Et, 37%); HRMS: Found 286.1801. Calcd. for C_15_H_18_NO_2_ 286.1807.

*Ethyl 2-(6-methoxyquinolin-4-yl)propanoate* (**10n**). Colourless oil, (58% purified yield); ν 1730 (C=O) cm^−1^; ^1^H-NMR: δ_H_ 1.16 (3Η, t, *J*_HH_ 7.0 Hz, -CΗ_2_CΗ_3_), 1.66 (3Η, d, *J*_HH_ 7.6 Hz, >CH-CH_3_), 3.95(3H, s, -OCH_3_), 4.15 (2H, q, *J*_HH_ 7.0 Hz, -O-CH_2_-), 4.39 (1H, q, *J*_HH_ 7.6 Hz, >CH-), 7.32 (1H, d, *J*_HH_ 2.5 Hz, H-5), 7.33 (1H d, *J*_HH_ 4.6 Hz H-3), 7.37 (1H, dd, *J*_HH_ 8.9 and 2.5 Hz, H-7), 8.04 (1H, d, *J*_HH_ 8.9 Hz, H-8), 8.73 (1H, d, *J*_HH_ 4.6 Hz, H-2); ^13^C-NMR: δ_C_ 14.00 (CH_3_), 17.22 (CH_3_), 41.38 (>CH-), 55.45 (OCH_3_), 61.22 (OCH_2_), 101.24 (5C), 119.44 (7C), 121.67 (3C), 127.71 (10C), 131.68 (8C), 144.48 (4C), 144.77 (9C), 147.84 (2C), 158.05 (6C), 173.63 (C=O); HRMS: Found 260.1286. Calcd. for C_15_H_18_NO_3_ 260.1287.

*Ethyl 2-(8-methoxyquinolin-4-yl)propanoate* (**10p**). Colourless oil, (54% purified yield); t_R_ 11.905 min (100%); ν 1729 (C=O) cm^−1^; ^1^H-NMR: δ_H_ 1.17 (3Η, t, *J*_HH_ 7.2 Hz, -OCH_2_-CH_3_), 1.66 (3Η, d, *J*_HH_ 7.6 Hz, >CH-CH_3_), 3.93 (3H, s, -OCH_3_), 4.14 (2H, q, *J*_HH_ 7.2 Hz, -O-CH_2_-), 4.39 (1H, q, *J*_HH_ 7.6 Hz, >CH-), 7.12 (1H, d, *J*_HH_ 7.0 and 1.0 Hz, H-7), 7.50 (1H d, *J*_HH_ 4.5 Hz H-3), 7.56 (1H, t, *J*_HH_ 7.0 Hz, H-6), 7.66 (1H, dd, *J*_HH_ 7.0 and 1.0 Hz, H-5), 8.98 (1H, d, *J*_HH_ 4.5 Hz, H-2); ^13^C-NMR: δ_C_ 13.94 (CH_3_), 17.69 (CH_3_), 41.58(>CH-), 56.15 (OCH_3_), 61.42 (OCH_2_), 108.29 (7C), 114.70 (5C), 119.93 (3C), 127.91 (6C), 128.00 (10C),138.65 (9C), 148.05 (4C), 150.02(2C), 155.21(8C), 173.24; HRMS: Found 260.1279. Calcd. for C_15_H_18_NO_3_ 260.1287.

*8-Methy-4-ethoxyquinoline* (**13**). Colourless oil, (8% purified yield); ν 1506 (-O-C-) cm^−1^; ^1^H-NMR: δ_H_ 1.47 (3Η, t, *J*_HH_ 7.1Hz, -OCH_2_-CH_3_ ), 2.79 (3Η, s, Αr-CH_3_), 4.08 (2H, q, *J*_HH_ 7.1 Hz, -O-CH_2_-), 6.57 (1H, d, *J*_HH_ 5.2 Hz, H-3), 7.32 (1H t, *J*_HH_ 6.7 Hz H-6), 7.48 (1H, dd, *J*_HH_ 1.4 and 6.7 Hz, H-7), 8.05 (1H, dd, *J*_HH_ 1.4 and 6.7 Hz, H-5), 8.71 (1H, d, *J*_HH_ 5.2 Hz, H-2); ^13^C-NMR: δ_C_ 14.41 (CH_3_), 18.55 (CH_3_), 63.95 (OCH_2_), 100.37 (3C), 119.81 (10C), 121.31 (5C), 125.11 (6C), 129.97 (7C), 136.33 (8C), 148.19 (2C), 150.09 (9C), 161.71 (4C); HRMS: Found 188.1080. Calcd. for C_12_H_14_NO 188.1075.

*8-(But-1-yl)-4-ethoxyquinoline* (**14**).Colourless oil, (9% purified yield); ν 1506 (-O-C-) cm^−1^; ^1^H-NMR: δ_H_ 0.93 (3Η, t, *J*_HH_ 7.1 Hz, -OCH_2_-CH_3_), 1.45 (2Η, sextet, −CH_2_-CH_3_), 1.54 (3H, t, *J*_HH_ 7.4 Hz, -O-CH_2_-CH_3_), 1.76 (2H, quintet, *J*_HH_ 7.4 Hz, -CH_2_-CH_2_-CH_2_-), 3.25 (2H, t, *J*_HH_ 7.4 Hz, Ar-CH_2_-), 4.20 (2H, q, *J*_HH_ 7.4 Hz, -O-CH_2_-), 6.67 (1H, d, *J*_HH_ 5.1 Hz, H-3), 7.39 (1H t, *J*_HH_ 6.9 Hz H-6), 7.52 (1H, dd, *J*_HH_ 1.4 and 6.9 Hz, H-7), 8.09 (1H, dd, *J*_HH_ 1.4 and 6.9 Hz, H-5), 8.74 (1H, d, *J*_HH_ 5.1 Hz, H-2); ^13^C-NMR: δ_C_ 14.16 (CH_3_), 14.51 (CH_3_), 22.84 (CH_2_), 31.46 (CH_2_), 32.82 (CH_2_), 64.00 (OCH_2_), 100.33 (3C), 119.67 (10C), 121.53 (5C), 125.14 (6C), 128.95979 (7C), 140.96 (8C), 147.86 (2C), 150.08 (9C), 161.73 (4C); HRMS: Found 230.1549. Calcd. for C_15_H_20_NO 230.1545.

### 3.2. Microbiology

#### 3.2.1. Determination of Susceptibility to Antimicrobial Agents

Minimum inhibitory concentrations (MICs) for all microorganisms with the exception of *H. pylori* were determined by microbroth dilution according to BSAC guidelines [33] in Mueller-Hinton broth. Microorganisms were obtained from the Sheffield Hallam University culture collection.

#### 3.2.2. Culture of *H. pylori*

The procedures employed for culture of *H. pylori* and analyses of anti-microbial activity were those previously described [34]. Cultures of *H. pylori* strains 3339 and 26695 [35] were obtained from Prof. David Kelly (University of Sheffield) and were obtained from *H pylori* patients with gastro-duodenal ulcers. These were bacterially typed as described [34] and cultured at 37 °C under microaerobic conditions (10% CO_2_, 3%O_2_ and 87% N_2_) in liquid Brucella broth (BB; BBL, USA) supplemented with foetal bovine serum (5% v/v; Invitrogen) [36].

#### 3.2.3. Determination of *H. pylori* Susceptibility to Antimicrobial Agents

Antimicrobial activity assays were performed using 24-well microplates. These were performed in multiple wells containing 0.1 mL of compound (diluted in DMSO) with 1.0 mL of early log phase *H. pylori* culture in Brucella broth with foetal bovine serum (as above). The plates were incubated at 37 °C under microaerobic conditions and bacterial growth was monitored twice daily at 600 nm using a microplate Multilabel Counter (Wallac Victor-1420, Finland). Compounds (1 mg) were dissolved in 10 μL of solvent (DMSO) to obtain a concentration of 100 mg/mL), and then serially diluted 100-fold into Brucella broth with foetal bovine serum to give concentrations of 10 to 10^−9^ mg/mL with 0.1 mL being added to each assay as detailed above. Cultures were incubated for a total of 7 days. MICs were determined by using serial dilution assays of the samples. The control mixtures in which the test compound was omitted indicated that the solvent, DMSO, did not have significant antimicrobial activity at the concentrations employed.

#### 3.2.4. Fixed Time-Point Measurements of Membrane Damage

Measurements of membrane integrity using the BacLight assay were made following a 10 min exposure to Ethyl 2-(quinolin-4-yl)-propanoates and comparator antibiotics at 4 X MIC as previously described in detail [37,38,39].

## 4. Conclusions

We have a simple method for the synthesis of novel 4-quinolylpropanoates **10a**–**p** as potential antimicrobial compounds. These 4-quinolylpropanoates have shown antimicrobial activities against a panel of microorganisms, but without Gram-specificity. However, the compounds showed potent antimicrobial activity against two strains of *H.* *pylori.* No evidence of membrane damage was observed in BacLight fixed time-point assays following exposure to any of the compound in this study (data not shown) suggesting an intracellular target. Quinoline derivatives have been associated with inhibition of DNA supercoiling in Mycobacterial species [40] and, although there is significant structural differences betweeen those molecules and the series presented in this study, this is a point of further investigation.
